# Efficient Thermally Evaporated Near-Infrared Perovskite Light-Emitting Diodes via Phase Regulation

**DOI:** 10.1007/s40820-025-01776-3

**Published:** 2025-05-22

**Authors:** Siwei He, Lanxin Qin, Zhengzheng Liu, Jae-Wook Kang, Jiajun Luo, Juan Du

**Affiliations:** 1 School of Physics and Optoelectronic Engineering, Hangzhou Institute for Advanced Study, University of Chinese Academy of Sciences, Hangzhou, 310024 People’s Republic of China; 2 Wuhan National Laboratory for Optoelectronics (WNLO) and School of Optical and Electronic Information, Huazhong University of Science and Technology (HUST), 1037 Luoyu Road, Wuhan, 430074 People’s Republic of China; 3 State Key Laboratory of Ultra-intense Laser Science and Technology, Shanghai Institute of Optics and Fine Mechanics, Chinese Academy of Sciences, Shanghai, 201800 People’s Republic of China; 4 Department of Flexible and Printable Electronics, LANL-JBNU Engineering Institute-Korea, Jeonbuk National University, Jeonju, 54896 Republic of Korea

**Keywords:** Vacuum-deposited perovskite, Near-infrared emission, Phase regulation, Co-evaporation, Electroluminescence

## Abstract

α-phase formamidinium lead triiodide (FAPbI_3_) has demonstrated extraordinary properties for near-infrared perovskite light-emitting diodes (NIR-PeLEDs). The vacuum processing technique has recently received increasing attention from industry and academia due to its solvent-free feature and compatibility with large-scale production. Nevertheless, vacuum-deposited NIR-PeLEDs have been less studied, and their efficiencies lag far behind those of solution-based PeLEDs as it is still challenging to prepare pure α-FAPbI_3_ by the thermal evaporation. Herein, we report a Cs-containing triple-source co-evaporation approach to develop the perovskite films. The addition of thermally stable Cs cation fills in the perovskite crystal lattice and eliminates the formation of metallic Pb caused by the degradation of FA cation during the evaporation process. The tri-source co-evaporation strategy significantly promotes the phase transition from yellow δ-phase FAPbI_3_ to black α-phase FACsPbI_3_, fostering smooth, uniform, and pinhole-free perovskite films with higher crystallinity and fewer defects. On this basis, the resulting NIR-PeLED based on FACsPbI_3_ yields a maximum EQE of 10.25%, which is around sixfold higher than that of FAPbI_3_-based PeLEDs. Our work demonstrates a reliable and effective strategy to achieve α-FAPbI_3_ via thermal evaporation and paves the pathway toward highly efficient perovskite optoelectronic devices for future commercialization.

**Supplementary Information:**

The online version contains supplementary material available at 10.1007/s40820-025-01776-3.

## Highlights

- α-phase formamidinium lead triiodide (FAPbI_3_) was prepared based on triple-source co-evaporation.
- A partial Cs-doping in the FAPbI_3_ can help with the suppression of the non-radiative recombination, elimination of the metallic Pb, improvement of spatial confinement.
- Near-infrared perovskite light-emitting diodes (NIR-PeLEDs) based on triple-source co-evaporated FACsPbI_3_ thin films achieved a maximum external quantum efficiency of 10.25%, which was around 6 times higher than that of FAPbI_3_-based NIR-PeLEDs.

**Supplementary Information:**

The online version contains supplementary material available at 10.1007/s40820-025-01776-3.

## Graphical abstract

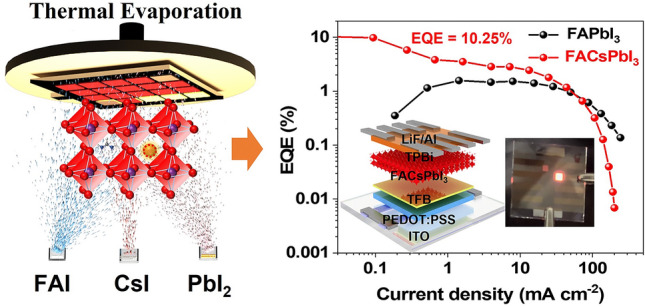

## Introduction

Metal halide perovskites have rapidly emerged as a revolutionary frontier in display and lighting owing to their fascinating properties including high color purity, excellent charge transport properties, tunable chromaticity, and cost-effectiveness [1–5]. Specially, near-infrared perovskite light-emitting diodes (NIR-PeLEDs) hold great potential for a variety of applications such as medical treatment, data storage, optical communications, hyperspectral imaging, night vision for surveillance, and automotive safety [6, 7].

With tremendous efforts of researchers, the external quantum efficiencies (EQEs) of NIR-PeLEDs have exceeded 23% within the last few years [2, 4], enabling them to be a strong competitor of the III-V NIR-LEDs. Up to date, the fabrication of formamidinium-based emitters such as FAPbI_3_ was regarded as the most successful approach to develop NIR-PeLEDs [2, 4, 7–9]. Nevertheless, most of the reported state-of-the-art NIR-PeLEDs were prepared via solution-based spin-coating, limiting their application in large-scale production. Inspired by the deposition of commercialized organic light-emitting diodes (OLEDs), thermal evaporation has been proposed as an alternative and effective technique to develop scalable PeLEDs [10]. Unlike solution-processing techniques, thermal evaporation can effectively avoid the problem of low solubility of some perovskite precursors. Besides, it can prevent the utilization of environmentally unfriendly organic solvents such as dimethylformamide (DMF) and dimethyl sulfoxide (DMSO). Furthermore, the process of thermal evaporation can be uniformly fabricated onto various substrates, paving a novel pathway for the heterogeneous integration of NIR-PeLEDs. Most importantly, thermal evaporation is compatible with the currently available OLED industry, thus lowering the initial investment and speeding up the commercialization of PeLEDs [3, 10–13].

The present researches on thermally evaporated PeLEDs are mainly focused on the green emission field [3, 11, 14–18]. The studies on thermally evaporated NIR-PeLEDs have not yet been investigated in-depth. Meanwhile, the efficiencies of thermally evaporated NIR-PeLEDs are still lagging far behind those of solution-processed NIR-PeLEDs [19]. Even though FAPbI_3_ has been widely reported in the solution-processed NIR-PeLEDs, it is believed that the direct reaction between formamidinium iodide (FAI) and lead iodide (PbI_2_) during the vapor deposition process usually causes the δ-phase and α-phase mixed FAPbI_3_ [20, 21]. This issue was demonstrated to be associated with the degradation of FA cation with the elevated evaporation temperature [22]. It is well known that the presence of hexagonal δ-phase limits the use of pure FAPbI_3_ as the active layer in both perovskite solar cells and PeLED [23–25]. The incorporation of mixed-cation (FA/MA/Cs) and/or mixed-halide (I/Br/Cl) has been frequently implemented into converting yellow δ-phase non-perovskite to a black α-phase in the solution-prepared FAPbI_3_ [26–29]. Compared with single cation FA-based perovskite, binary A-site cation FA/Cs-based perovskite has led to more efficient and stable perovskite due to the higher thermal stability of Cs cation [30]. Nevertheless, the employment of mixed-cation and mixed-halide stoichiometries in the thermal evaporation is technically difficult because of the complication of fabricating multiple perovskite precursors and additives simultaneously [21].

Herein, we proposed a partial replacement of FA cation by Cs cation to stabilize the perovskite crystal structure and prepare α-phase FACsPbI_3_ thin film by simultaneously thermal co-evaporation of three source, *i.e.*, FAI, PbI_2_, and CsI, for the application of NIR-PeLEDs. Subsequently, we systematically studied how the triple-source co-evaporation forms pure black α-phase FACsPbI_3_ in the vacuum deposition process. We demonstrated that alloying partial Cs into FAPbI_3_ can lead to not only the enhancement of the α-phase crystallinity but also the improvement of surface morphologies. Meanwhile, the FACsPbI_3_ thin films exhibited reduced nanocrystal size and suppressed trap densities, resulting in a significant enhancement of spatial confinement and radiative recombination. Leveraging these benefits, we fabricated the NIR-PeLEDs based on thermally evaporated FACsPbI_3_ thin films and achieved a maximum EQE value of 10.25%, which was around six times higher than that of FAPbI_3_-based NIR-PeLEDs.

## Experimental Section

### Materials

Formamidinium iodide (FAI, > 99.99%) was purchased from GreatCell Solar. Lead iodide (PbI_2_, 99.999%), cesium iodide (CsI, 99.999%), lithium fluoride (LiF, > 99.99%), chlorobenzene (99.8%, anhydrous) were purchased from Sigma-Aldrich. Poly(ethylene dioxythiophene)/polystyrene sulfonate (PEDOT:PSS, AI 4083) was purchased from Xi’an Yuri Solar Co. Ltd. Poly(9,9-dioctylfluoreneyl-2,7-diyl)-alt-(4,4’-(N-(4-butylphenyl)-diphenylamine) (TFB) and 1,3,5-tris(1-phenyl-1H-benzimidazol-2-yl)benzene (TPBi) were purchased from Xi’an Polymer Light Technology Corp.

### Preparation of Perovskite Light-Emitting Diodes

#### Preparation of Perovskite Films

For the perovskite deposition, a thermal evaporation integrated into an N_2_ glovebox was used. The system was firstly vacuumed to a base pressure of 1.0 × 10^–5^ Torr before the source was heated. Three quartz crystal microbalances (QCMs) were employed to measure the rate of each source independently. Prior to deposition, the deposited rate and thickness of PbI_2_ and FAI were calibrated using QCM and Alpha-step profiler. For the evaporation of FAPbI_3_, FAI and PbI_2_ were kept in the independent crucibles and slowly heated to a target rate of 0.1 and 0.05 Å s^−1^, separately. For the evaporation of FACsPbI_3_, the evaporated rates of FAI, PbI_2_, and CsI were maintained at 0.1, 0.05, and 0.02 Å s^−1^, respectively. Due to the complicated evaporated behavior of FAI, we kept the FAI source at 55 °C for 30 min before gradually heating to the sublimation temperature (~ 100–120 °C for FAI) to avoid abrupt evaporation and decomposition. At the same time, the vacuum pressure of the system was increased to ~ 5.0 × 10^–4^ Torr. It should be noted that the substrates holder did not rotate during the evaporation.

#### Preparation of Perovskite Light-Emitting Diodes

The patterned indium tin oxide (ITO) glass substrates were sequentially sonicated in ethanol and water for 20 min. Afterward, the substrates were treated with UV-ozone for 15 min. The PEDOT:PSS was spin-coated onto the pre-cleaned ITO-patterned glass substrate at 2000 r min^−1^ for 30 s followed by annealing at 150 °C for 30 min. The PEDOT:PSS coated substrates were then transferred to the N_2_ glovebox. In the glovebox, a TFB solution dissolved in chlorobenzene at a concentration of 5 mg mL^−1^ was spin-coated onto the PEDOT:PSS film at 3000 r min^−1^ for 30 s. The TFB layer was baked at 150 °C for 30 min. After that, these samples were transferred to a thermal evaporation chamber integrated with a glovebox for the deposition of perovskite emitting layer. The emitting layer of FAPbI_3_ and FACsPbI_3_ was deposited by co-evaporation of FAI, PbI_2_, and CsI in individual crucibles. The thickness of emission layer was around 20 nm. After that, TPBi (40 nm), LiF (1 nm), and aluminum (90 nm) were thermally evaporated onto the perovskite films.

### Characterizations

#### Characterization of Perovskite Films

X-ray diffraction (XRD) spectra of perovskite films were obtained by Smart Lab 9KW multifunctional rotating-anode X-ray diffractometer using Cu Kα radiation. The absorption spectra of the perovskite films were analyzed using an ultraviolet–visible (UV–Vis–near-IR) spectrophotometer (Shimadzu Instruments, SolidSpec-3700). The steady-state photoluminescence (PL) spectra were tested using a fluorescence spectrometer (Horiba, QuantaMaster 8000). Time-resolved photoluminescence (TRPL) decay was measured using a Horiba Fluorolog-QM series. A single-mode 440 nm pulsed diode laser (pulse width of 75 ps; average power: 2.4 mW) was used as an excitation source. X-ray photoelectron spectroscopy (XPS) and ultraviolet photoelectron spectroscopy (UPS) of perovskite films were analyzed using X-ray photoelectron spectrometer (AXIS-ULTRA DLD-600W, Shimadzu) system. The transient absorption (TA) measurement with similar pump fluences was conducted by a commercial equipment (Ultrafast system, Helios fire) with a 400 nm wavelength pulse laser. The topography and surface morphology were characterized with an atomic force microscope (AFM, Jupiter XR, Oxford Instruments Asylum Research, Inc.) and field-emission scanning electron microscope (FE-SEM, FEI Nova NanoSEM 450).

#### Characterization of Perovskite Light-Emitting Diodes

The active area of PeLED devices was 4 mm^2^ which was defined by the overlapping area of ITO and Al electrodes. The electroluminescence (EL) performance including current density–voltage–radiance characteristics, external quantum efficiency, and EL spectra of PeLEDs were collected by a computer-controlled integrated system, consisting of a programmable Keithley 2400 source meter and XPQY-EQE (Guangzhou XiPu Optoelectronics Technology) equipped with an integrating sphere and a photodetector. The measurement system was calibrated by halogen lamps metered by the National Institute of Standards and Technology (NIST) and installed inside an N_2_-filled glovebox.

## Results and Discussion

### Preparation of Thermally Evaporated Perovskites

**Fig. 1:**
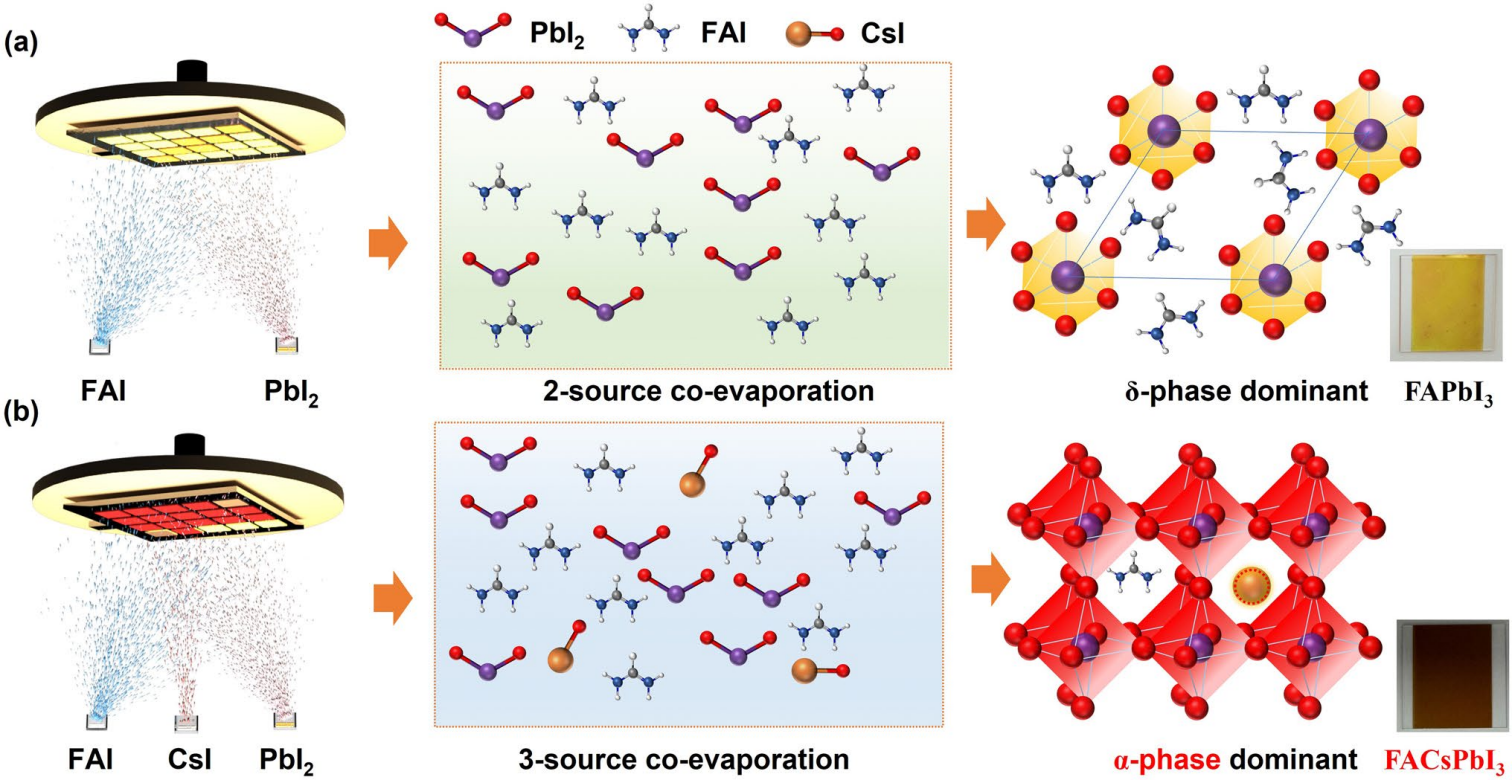
Preparation of thermally evaporated perovskite thin films. **a** Dual-source co-evaporation of FAPbI_3_ formed via FAI and PbI_2_, and **b** triple-source co-evaporation of FACsPbI_3_ formed via FAI, CsI, and PbI_2_

In this work**,** we deposited the dual-source evaporated FAPbI_3_ and triple-source evaporated FACsPbI_3_ perovskite thin films on a steady substrate to develop spatially gradient components according to previously reported publications [11, 18]. Utilizing a fixed substrate simplifies maintaining stoichiometric consistency, which is critical for high-throughput optimization process. However, substrate rotation introduces challenges in maintaining precious compositional control. In Fig. S1, the as-deposited spatially gradient FAPbI_3_ and FACsPbI_3_ thin films obtained via thermal evaporation were illustrated. There was a gradient color distribution in the FAPbI_3_ thin films, and most FAPbI_3_ thin films exhibited a yellowish color, while all the FACsPbI_3_ thin films showed a brown color. Unlike the easy deposition of organic materials in OLEDs, the evaporated process of perovskite is complicated, involving precursor evaporation accompany with decomposition, chemical reaction, nucleus formation, and crystal growth. Figure 1a illustrates the preparation process of FAPbI_3_ thin films based on the direct reaction between FAI and PbI_2_ using the thermal evaporation process. During the evaporation of FAI, it can be easily degraded to hydrogen cyanide and hydrogen iodide, which further react to form 1.3.5-sym-triazine and ammonia. With increasing temperature, FA may degrade and leave the crystal lattice, remaining the unreacted PbI_2_ and metallic Pb. This leads to a yellowish color in the as-deposited FAPbI_3_ thin film, indicating the presence of δ-phase FAPbI_3_ (Fig. 1a). Typically, the δ-phase FAPbI_3_ has a hexagonal structure that leads to non-photoactivity [20, 31].To address this problem, co-evaporation of FAI, PbI_2_, and CsI was proposed to convert the yellow δ-phase to the black α-phase and stabilize the perovskite phase. During the evaporation, the thermally stable Cs cation can fill the empty A-cation sites and stabilize the crystal structure of FAPbI_3_ (Fig. 1b). The photograph of the as-deposited FACsPbI_3_ thin film exhibited a different brown color from that of FAPbI_3_ thin film. This can be ascribed to the dominant α-FAPbI_3_ phase in the FACsPbI_3_ thin film, which has an ordered cubic structure.

### Fundamental Photoelectronic Properties of Perovskites

**Fig. 2:**
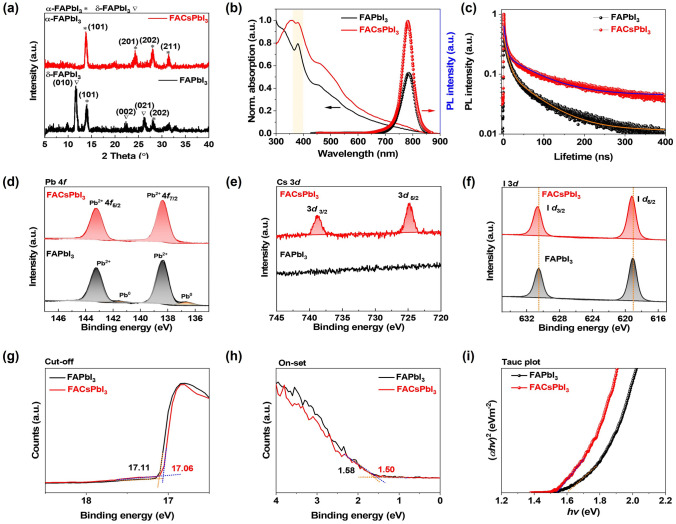
**a** XRD patterns, **b** UV–Vis absorption and PL spectra, **c** time-resolved PL spectra and corresponding fitting spectra of FAPbI_3_ and FACsPbI_3_. XPS spectra of the **d** Pb 4*f*, **e** Cs 3*d*, and **f** I 3*d*, UPS spectra at the **g** cutoff region, and **h** onset region, **i** Tauc plot of FAPbI_3_ and FACsPbI_3_

To understand the crystalline phases formed in the FAPbI_3_ and FACsPbI_3_ thin films, we conducted X-ray diffraction (XRD) measurements. As shown in Fig. 2a, the diffraction peaks of the as-evaporated FAPbI_3_ at 11.7° and 14.0° evidenced the coexistence of δ-phase and α-phase FAPbI_3_ [23, 26, 32]. Notably, the δ-FAPbI_3_ phase at 11.7° was almost absent in the FACsPbI_3_ thin film, confirming the conversion to the α-FAPbI_3_ phase after co-evaporation with CsI. Furthermore, Fig. 2b compares the UV–Vis absorption spectra of FAPbI_3_ and FACsPbI_3_ thin films. Both FAPbI_3_ and FACsPbI_3_ thin films had a characteristic absorption edge at around 800 nm, ascribed to the absorption edge of the α-phase FAPbI_3_ [23]. It was noted that there was a sharp absorption peak at 380 nm which as attributed to the δ-FAPbI_3_ phase [21]. However, no obvious δ-phase signal was observed in the FACsPbI_3_ thin films. The photoluminescence (PL) spectra of the co-evaporated FAPbI_3_ and FACsPbI_3_ are also shown in Fig. 2b. The PL peak of the FAPbI_3_ thin film was located at a wavelength of 784 nm with a full-width at half-maximum (FWHM) of 55 nm. In contrast, the PL peak of the FACsPbI_3_ thin film was at 782 nm with a narrower FWHM of 45 nm. The narrower PL emission observed in the triple-source evaporated FACsPbI_3_ thin films may be an indication of improved monodispersity and reduced defects [33]. In addition, the PL quantum yield (PLQY) of the FACsPbI_3_ thin film reached ~ 26%, which is more than eight times as high as that of the FAPbI_3_ thin film (PLQY ~ 3%). The PL intensity of the FACsPbI_3_ thin film was much higher than that of the FAPbI_3_ thin film, suggesting an enhanced PL emission and reduced non-radiative recombination in the FACsPbI_3_ thin film. To confirm this, time-resolved PL (TRPL) measurement was performed to assess the carrier recombination dynamics of FAPbI_3_ and FACsPbI_3_ thin films. The TRPL spectra were fitted by bi-exponential decay (Eq. S1), and the average PL lifetime was determined using Eq. S2. The fitting details are summarized in Table S1. As illustrated in Fig. 2c, FACsPbI_3_ thin film showed a longer average carrier lifetime of 51.02 ns than that of FAPbI_3_ thin film with an average lifetime of 48.55 ns, revealing the reduced defects and non-radiative recombination. XPS measurements were further conducted to investigate the detailed chemical states of the FAPbI_3_ and FACsPbI_3_ thin films (Fig. S2). In Fig. 2d, there were two main peaks Pb 4*f*_5/2_ and Pb 4*f*_7/2_ at 143.1 and 138.3 eV, respectively. Notably, the doublet peaks at around 141.3 and 136.4 eV corresponding to the metallic Pb states were obvious in the FAPbI_3_ thin film. The presence of a large substantial of atomic Pb was likely to be Pb-interstitial defects in the perovskite lattice of FAPbI_3_ [34, 35]. The atomic Pb species can serve as trap states for the non-radiative recombination and would greatly undermine the optoelectronic properties of PeLEDs. In contrast, the metallic Pb state peaks were not observed in the triple-source evaporated FACsPbI_3_. As shown in Fig. 2e, no Cs 3*d* peaks were detected from FAPbI_3_ thin film, while they were observed from FACsPbI_3_ thin film, confirming the successful addition of Cs via triple-source co-evaporation. Furthermore, we calculated the ratio of Cs/Pb via the integrated areas of XPS spectra for Pb 4*f* and Cs 3*d* peaks. The ratio of Cs/Pb was determined to be 12% for the optimized FACsPbI_3_ thin film. In the I 3*d* core-level spectra, both FAPbI_3_ and FACsPbI_3_ exhibited two dominant I 3*d*_3/2_ and I 3*d*_5/2_ peaks (Fig. 2f). Compared with FAPbI_3_, the I 3*d*_3/2_ and I 3*d*_5/2_ peaks of FACsPbI_3_ slightly shift toward higher binding energy, which may be ascribed to the stronger bond energies between Pb and I [36].

The energy levels of FAPbI_3_ and FACsPbI_3_ were estimated by ultraviolet photoelectron spectroscopy (UPS), as illustrated in Fig. S3. The cutoff energy (*E*_cutoff_) and onset energy (*E*_onset_) of FAPbI_3_ and FACsPbI_3_ are shown in Fig. 2g, h. The optical bandgap (*E*_g_) values of FAPbI_3_ and FACsPbI_3_ thin films were fitted from the Tauc plots in Fig. 2i. The *E*_g_ values of the FAPbI_3_ and FACsPbI_3_ were 1.60 and 1.54 eV, respectively. The valance band maximum (*E*_VBM_), Fermi level (*E*_Fermi_), and conduction band minimum (*E*_CBM_) values of FAPbI_3_ and FACsPbI_3_ were calculated using Eqs. S3-S5 and are summarized in Table S2. The *E*_CBM_ values of FAPbI_3_ and FACsPbI_3_ were determined to be − 5.69 and − 5.66 eV, respectively. Compared with FAPbI_3_ thin films, there was a small upshift of *E*_CBM_ in the FACsPbI_3_ thin films, leading to better hole transportation.

**Fig. 3:**
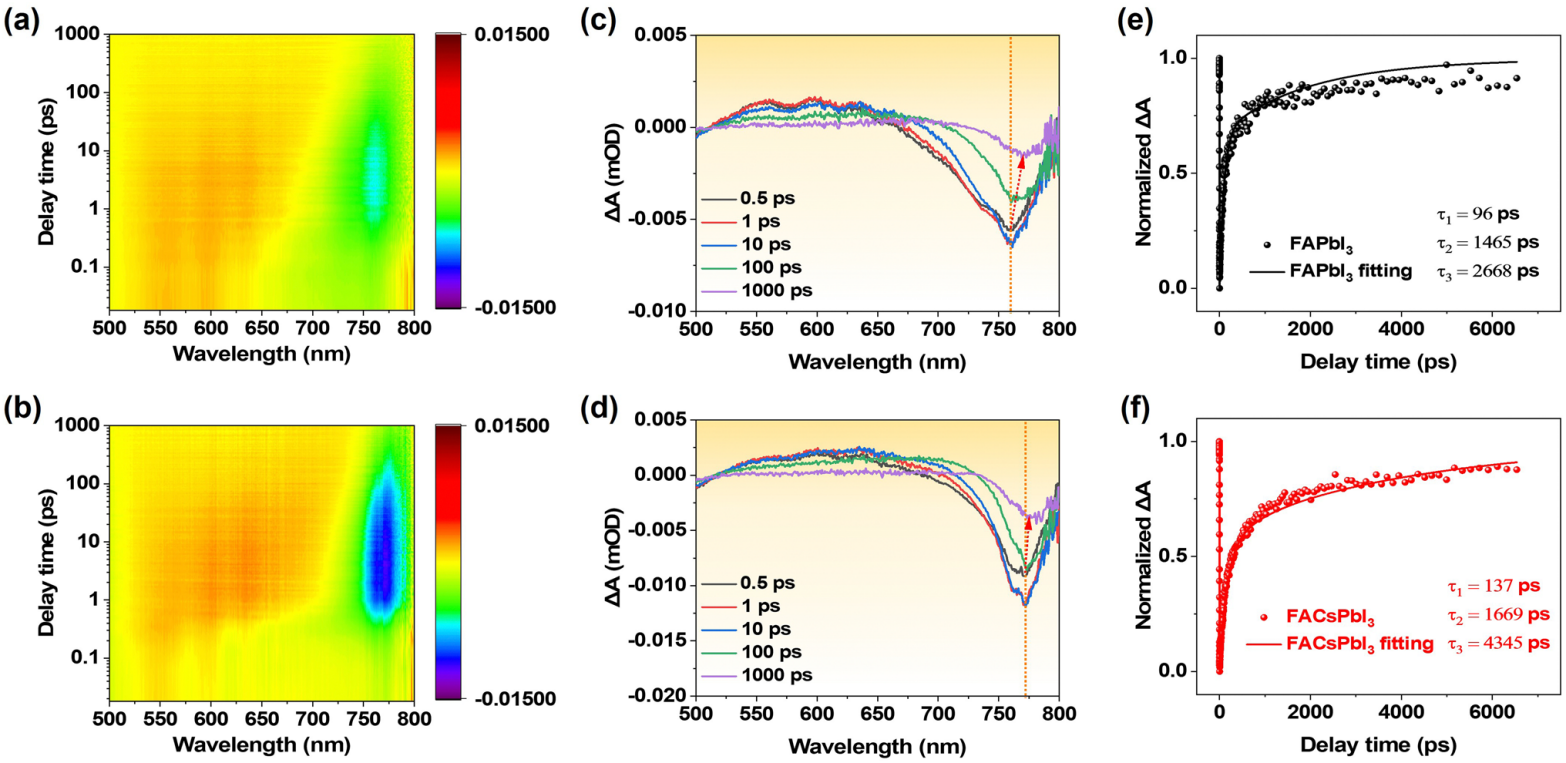
Time-wavelength-dependent TA contour maps of **a** FAPbI_3_ and **b** FACsPbI_3_. TA spectra at selected timescales of **c** FAPbI_3_ and **d** FACsPbI_3_. Kinetic traces at a probing wavelength of ~ 770 nm for **e** FAPbI_3_ and **f** FACsPbI_3_

Transient absorption (TA) spectroscopy was applied to study the carrier dynamics in the FAPbI_3_ and FACsPbI_3_ thin films. The two-dimensional TA spectra as a function of wavelength and pump–probe delay time for the FAPbI_3_ and FACsPbI_3_ thin films are illustrated in Fig. 3a, b. The TA contour maps of both the FAPbI_3_ and FACsPbI_3_ thin films exhibited a distinct ground-state bleaching (GSB) peak at around 770 nm and the photoabsorption (PA) positive band at 520–700 nm, which are in agreement with earlier publications [2, 37]. By comparing the GSB peak at around 770 nm between FAPbI_3_ and FACsPbI_3_, it was noted that the negative peak intensity of the FACsPbI_3_ was greatly enhanced. For better comparison, TA spectra at selected timescales of the FAPbI_3_ and FACsPbI_3_ thin films are extracted in Fig. 3c, d. Once the perovskite thin films absorb photons, the photoexcited carriers owing to the bandgap renormalization effect would relax to the lowest energy sites, resulting in a redshift of the GSB [38–40]. After a photoexcitation time of 100 ps, it was obvious that the FAPbI_3_ showed a greater redshift of ~ 5 nm than that of FACsPbI_3_ (redshift: ~ 2 nm). The reduced redshift of FACsPbI_3_ thin film suggested a flatter energy landscape and reduced tail states below the bandgap. Furthermore, as shown in Fig. 3e, f, TA traces as a function of delay time at a probing wavelength of ~ 770 nm were extracted and fitted with a tri-exponential function. The fast decay (*τ*_1_) is attributed to the relaxation of photogenerated hot carriers. A higher carrier concentration can slow hot carrier cooling due to screened electron–phonon interactions [41]. The intermediate decay (*τ*_2_) corresponds to the carrier trapping of defects. Compared with FAPbI_3_, FACsPbI_3_ showed a prolonged *τ*_2_, suggesting reduced carrier quenching sites. Consequently, the bleach signal for the FACsPbI_3_ thin film exhibited a higher fraction of long-lived component (longest decay *τ*_3_ = 4345 ps) than that of the FAPbI_3_ thin film (longest decay *τ*_3_ = 2668 ps), which was responsible for the recombination of photoexcited species [40]. The higher fraction of the long-lived component (*τ*_3_) in FACsPbI_3_ suggests a greater contribution from radiative recombination, which is also consistent with the enhanced photoluminescence intensity, leading to higher efficiency and better luminescence properties [42].

### Morphology Studies of Perovskites

**Fig. 4:**
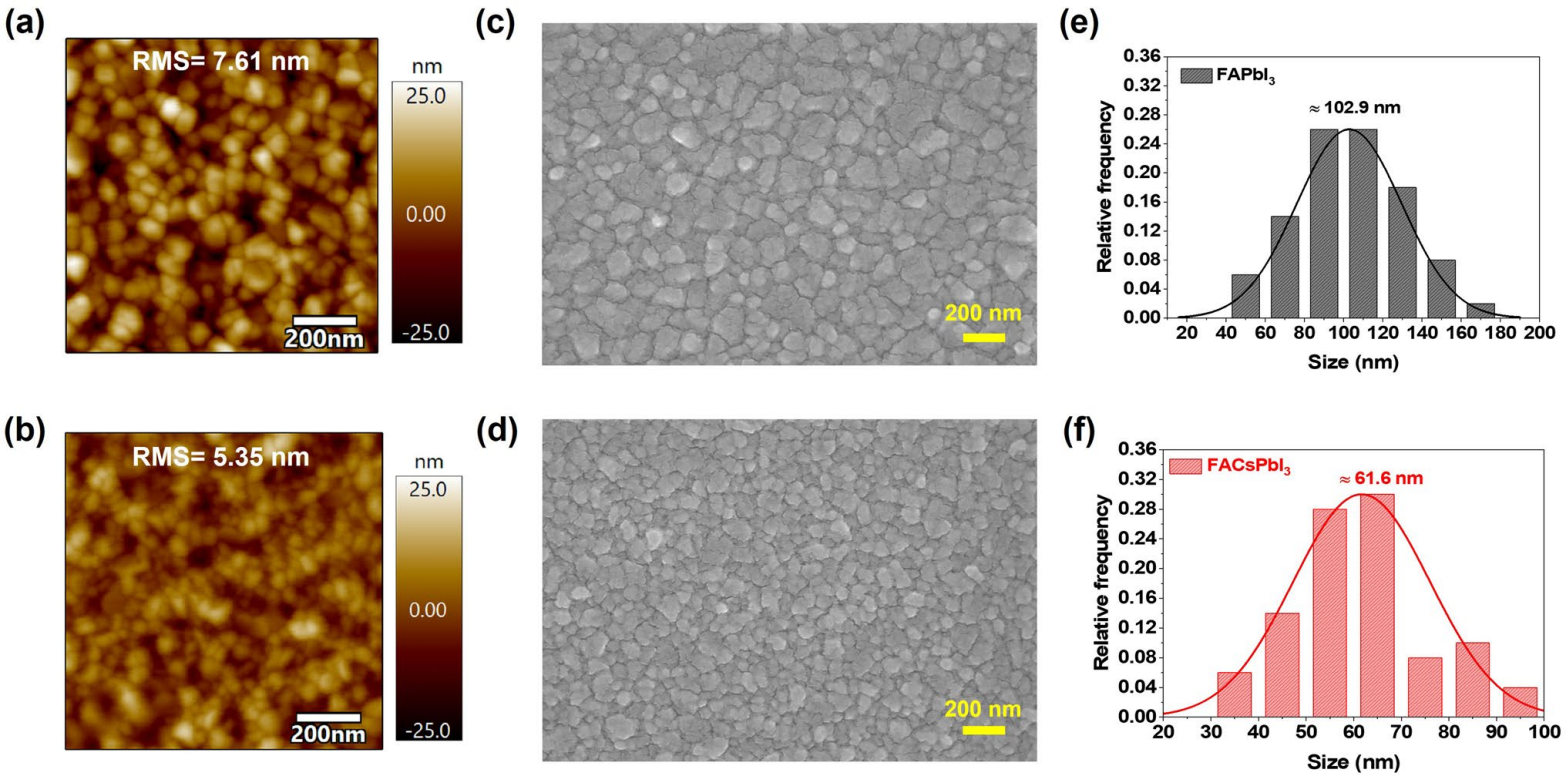
AFM micrographs of **a** FAPbI_3_ and **b** FACsPbI_3_. FE-SEM images of **c** FAPbI_3_ and **d** FACsPbI_3_. The crystal size distribution of **e** FAPbI_3_ and **f** FACsPbI_3_

To investigate the effects of Cs addition to the FAPbI_3_ on the topographies and surface morphologies, we conducted atomic force microscope (AFM) and field-emission scanning electron microscope (FE-SEM) measurements of evaporated FAPbI_3_ and FACsPbI_3_ on indium tin oxide (ITO) substrate. As shown in Fig. 4a, b, the FACsPbI_3_ exhibited a lower surface roughness with a root-mean-square (RMS) value of 5.35 nm compared with that of FAPbI_3_ at 7.61 nm. The lower roughness of FACsPbI_3_ could form better contact with the hole transport layer, enabling better hole injection and transportation. Furthermore, the FACsPbI_3_ perovskite film had a smaller nanograin compared with that of FAPbI_3_ (Fig. 4c, d). As illustrated in Fig. 4e, f, the resulting evaporated FACsPbI_3_ film had a smaller nanocrystal size of ~ 61.6 nm than that of FAPbI_3_ with an average nanocrystal size of ~ 102.9 nm. For better comparison, we also deposited the perovskite thin films on the PEDOT:PSS/TFB hole transport layers (HTLs). As illustrated in Fig. S4, the resulting evaporated FACsPbI_3_ exhibited much more compact and dense morphology compared with FACsPbI_3_. Besides, FACsPbI_3_ film still had a smaller nanocrystal size of ~ 77.7 nm than that of FAPbI_3_ with an average nanocrystal size of ~ 135.6 nm. The partial substitution of smaller Cs may eliminate the large crystallite of δ-phase FAPbI_3_ and suppress the smaller remnant inclusions that acted as the potential defects, leading to intrinsic spatial confinement in FACsPbI_3_ [29].The enhanced spatial confinement effect of FACsPbI_3_ was significantly beneficial for the application of PeLEDs.

### Device Performance of PeLEDs

**Fig. 5:**
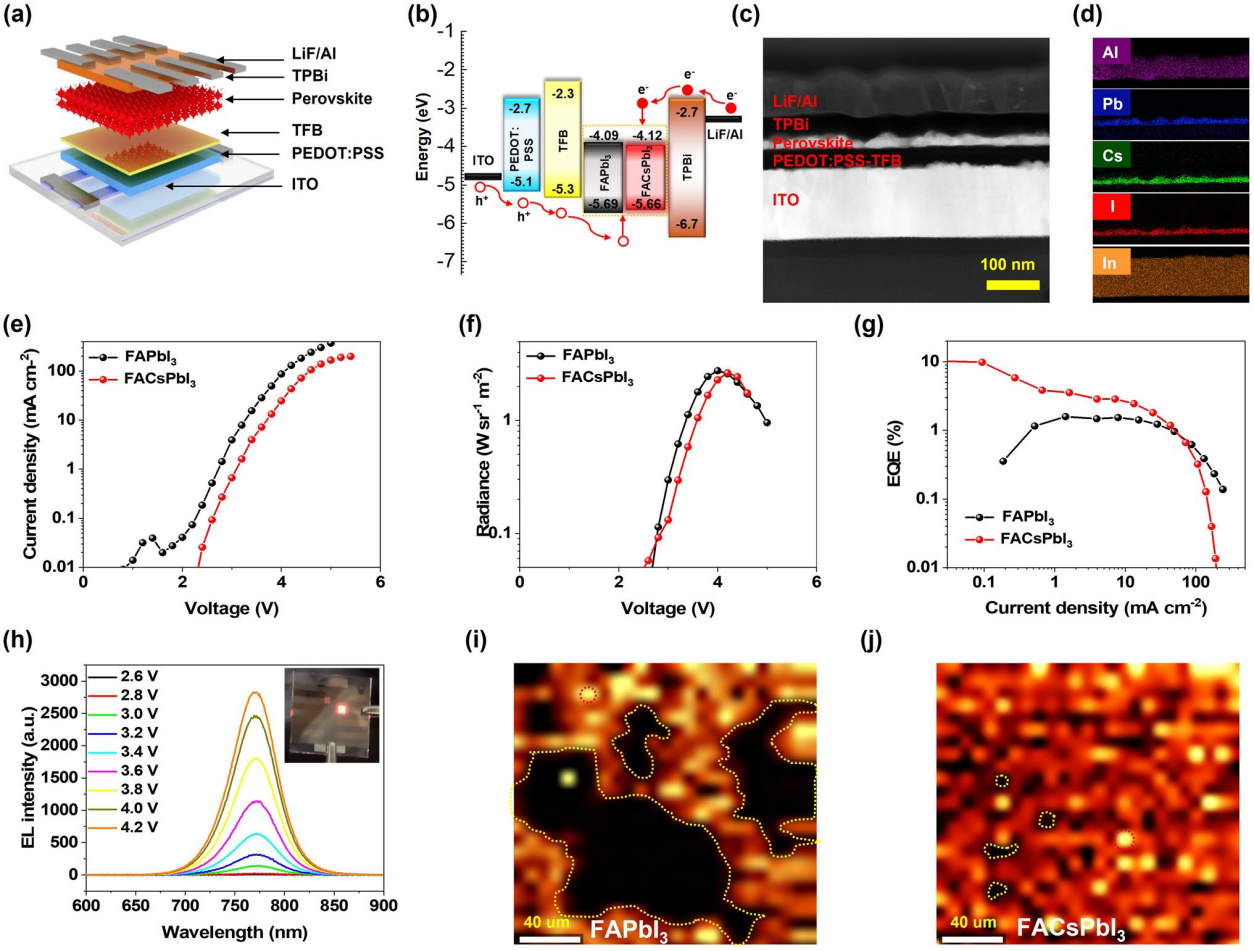
**a** Device structure, and **b** energy levels of PeLEDs. **c** A STEM-HAADF image of cross-section a full PeLEDs based on FACsPbI_3_. **d** EDS element mapping of Al, Pb, Cs, I, and In of the PeLEDs. Electroluminescence performance: **e** current density–voltage curves, **f** radiance–voltage curves, **g** EQE–current density of FAPbI_3_ and FACsPbI_3_. **h** EL spectra of FACsPbI_3_-based PeLEDs at different operating voltages. The inset shows a working device at a voltage of 4 V. Electroluminescence mapping of **i** FAPbI_3_- and **j** FACsPbI_3_-based PeLEDs device at a voltage of 6 V. The yellow dash region and red dash region denoted dark and emission region, respectively

We fabricated FAPbI_3_- and FACsPbI_3_-based NIR-PeLEDs with the following device configuration: indium tin oxide (ITO) as an anode, poly(ethylene dioxythiophene)/polystyrene sulfonate (PEDOT:PSS) as hole injection layer, poly(9,9-dioctylfluoreneyl-2,7-diyl)-alt-(4,4′-(N-(4-butylphenyl)-diphenylamine) (TFB) as hole transport layer, evaporated perovskite as emission layer, 1,3,5-tris(1-phenyl-1H-benzimidazol-2-yl)benzene (TPBi) as electron transport layer and LiF/Al as cathode (Fig. 5a). The corresponding energy-level diagram of PeLEDs is illustrated in Fig. 5b. To observe the crystal quality and interface, high-resolution scanning transmission electron microscopy (STEM) was performed on the thin cross-section lamella of the PeLED device. The lamellas of devices were prepared via a focused ion beam (FIB) (Fig. S5). As shown in Fig. 5c, FAPbI_3_- and FACsPbI_3_-based PeLED devices were analyzed using STEM under the high-angle annular dark-field (HAADF) mode. Based on the STEM images of the FAPbI_3_- and FACsPbI_3_-based PeLED devices in Fig. S6, the thickness of PEDOT:PSS/TFB hole transport layer was determined to be 40 ~ 50 nm. The thickness of FAPbI_3_ and FACsPbI_3_ emitting layers was determined to be around 20 nm. The thickness of the TPBi electron transport layer was ~ 40 nm. Figure 5d illustrates the energy-dispersive X-ray spectrometer (EDS) elemental mapping of In, I, Cs, Pb, and Al in the FACsPbI_3_-based PeLEDs, demonstrating a uniform distribution of the elements in the PeLED device. Compared with the EDS mapping of FAPbI_3_ device in Fig. S6, the only difference observed in the EDS mapping was the Cs element in the FACsPbI_3_ emission layer. The current density–voltage and radiance–voltage curves of PeLEDs are shown in Fig. 5e, f. The current density in the FAPbI_3_-based device was observed to be higher than that of FACsPbI_3_-based device (Fig. 5e). The higher current density of FAPbI_3_-based device may be ascribed to the larger grain size of FAPbI_3_ compared with FACsPbI_3_, leading to a smaller number of grain boundaries in FAPbI_3_ thin films [43]. The FAPbI_3_- and FACsPbI_3_-based devices had a similar radiance of 2.76 and 2.64 W Sr^−1^ m^−2^, respectively. As illustrated in Fig. 5g, the EQE value of the device based on FACsPbI_3_ reached a maximum of 10.25%, which was approximately 6.5-fold higher than that of the device based on FAPbI_3_ (EQE = 1.58%). To address the advantage of the high-throughput optimization of fixing the substrate during the evaporation, we also conclude EQE value and wavelength peak distribution of the devices in the different deposited regions. As shown in Fig. S7, the FACsPbI_3_-based devices showed overall enhancements in the efficiency compared with their FAPbI_3_-based counterparts. The efficiency enhancement could be attributed to the enhanced α-phase crystallinity and reduced trap density of FACsPbI_3_. To provide a comparison, we summarized the performance of state-of-the-art NIR-PeLEDs based on both solution-based process and thermal evaporation (Table S3). The optimal device based on evaporated α-phase FACsPbI_3_ represents one of the best devices for a thermally evaporated NIR-PeLEDs ever reported so far. In order to further investigate the carrier modulation effect, space-charge-limited current (SCLC) analysis was performed. The hole-only devices with a configuration of ITO/PEDOT:PSS/TFB/Perovskite/MoO_3_/Ag and the electron-only devices with a structure of ITO/TPBi/Perovskite/TPBi/LiF/Al based on FAPbI_3_ and FACsPbI_3_ were fabricated. The trap density (*n*_t_) was calculated from the current density–voltage curves using the Mott–Gurney equation [44, 45]:

3 $$ n_{t} = \, 2\varepsilon_{0} \varepsilon V_{{{\text{TFL}}}} /eL^{2} $$

where *ε*_0_ and *ε* are the vacuum permittivity and relative permittivity, respectively. Here, *e* represents the elementary charge and *L* is the thickness of perovskite films. The V_TFL_ is the trap-filling limit voltage, which can be estimated by the current density–voltage curves. As shown in Fig. S8, the fitted V_TFL_ values of the hole-only device based on FAPbI_3_ and FACsPbI_3_ were 0.95 and 0.83 V, respectively. Similarly, the V_TFL_ values of the electron-only device based on FAPbI_3_ and FACsPbI_3_ were 1.02 and 0.94 V, respectively. Therefore, the corresponding *n*_t_ values of the hole-only device were estimated to be 5.25 × 10^18^ and 4.59 × 10^18^ cm^−3^, respectively. Meanwhile, the *n*_t_ of the electron-only device was calculated to be 5.64 × 10^18^ and 5.19 × 10^18^ cm^−3^, respectively. The decreased *n*_t_ value strongly verified the reduced trap densities of the FACsPbI_3_ films. As shown in Fig. 5h, the electroluminescence (EL) peak of the device based on FACsPbI_3_ was located at ~ 770 nm. There was no obvious peak shift with the increasing driving voltages. EL mapping was conducted to assess the EL degradation of FAPbI_3_- and FACsPbI_3_-based PeLEDs. As shown in Fig. 5I, j, a confocal fluorescence microscope was used to measure the EL mapping of the devices at a voltage of 6 V. Due to the photoinactive δ-phase and relatively poor morphologies of FAPbI_3_, there was a greatly enlarged dark area (yellow dash region) in the FAPbI_3_-based device during working, which was likely due to the deterioration of the emission layer [46, 47] (Fig. 5i). On the contrary, the FACsPbI_3_-based device exhibited much higher overall radiative intensities and more homogeneous radiative distributions (Fig. 5j).

## Conclusions

In conclusion, we comprehensively studied the effect of A-site Cs substitution on the optoelectronic properties of FAPbI_3_ thin films in vapor deposition. Through triple-source co-evaporation, α-phase FACsPbI_3_ thin films with PL located at 782 nm and a nanocrystal size of ~ 61 nm were prepared. Detailed and systematic characterizations confirmed that partially replacing FA with Cs in the FAPbI_3_ can help with the suppression of the non-radiative recombination, reduction of the nanocrystal size, elimination of the metallic Pb, and enhancement of the surface morphology of FAPbI_3_ thin films. As a result, the NIR-PeLEDs based on α-phase FACsPbI_3_ achieved a maximum EQE of 10.25%, significantly surpassing that of NIR-PeLEDs based on FAPbI_3_ (EQE = 1.58%). This work offers a feasible and reliable route to improve the performance of NIR-PeLEDs. Furthermore, it can be expected that this work will also inspire the development of scalable perovskite-based optoelectronic devices.

## Supplementary Information

Below is the link to the electronic supplementary material.

Supplementary file1 (DOCX 1553 KB)

## References

[1] X. Zhao, Z.-K. Tan, Large-area near-infrared perovskite light-emitting diodes. Nat. Photonics **14**(4), 215–218 (2019). 10.1038/s41566-019-0559-3

[2] Y. Sun, L. Ge, L. Dai, C. Cho, J.F. Orri et al., Bright and stable perovskite light-emitting diodes in the near-infrared range. Nature **615**(7954), 830–835 (2023). 10.1038/s41586-023-05792-4 36922588 10.1038/s41586-023-05792-4

[3] J. Li, P. Du, Q. Guo, L. Sun, Z. Shen et al., Efficient all-thermally evaporated perovskite light-emitting diodes for active-matrix displays. Nat. Photonics **17**(5), 435–441 (2023). 10.1038/s41566-023-01177-1

[4] Y. Liu, C. Tao, Y. Cao, L. Chen, S. Wang et al., Synergistic passivation and stepped-dimensional perovskite analogs enable high-efficiency near-infrared light-emitting diodes. Nat. Commun. **13**(1), 7425 (2022). 10.1038/s41467-022-35218-0 36460647 10.1038/s41467-022-35218-0 PMC9718757

[5] S. He, H.B. Lee, K.-J. Ko, N. Kumar, J.-H. Jang et al., Optical engineering of FAPbBr_3_ nanocrystals via conjugated ligands for light-outcoupling enhancement in perovskite light-emitting diodes. Adv. Opt. Mater. **11**(17), 2300486 (2023). 10.1002/adom.202300486

[6] Y. Liu, F. Di Stasio, C. Bi, J. Zhang, Z. Xia et al., Near-infrared light emitting metal halides: materials, mechanisms, and applications. Adv. Mater. **36**(21), 2312482 (2024). 10.1002/adma.202312482 10.1002/adma.202312482 38380797

[7] M. Vasilopoulou, A. Fakharuddin, F.P. García de Arquer, D.G. Georgiadou, H. Kim et al., Advances in solution-processed near-infrared light-emitting diodes. Nat. Photonics **15**(9), 656–669 (2021). 10.1038/s41566-021-00855-2

[8] Z.-L. Tseng, L.-C. Chen, L.-W. Chao, M.-J. Tsai, D. Luo et al., Aggregation control, surface passivation, and optimization of device structure toward near-infrared perovskite quantum-dot light-emitting diodes with an EQE up to 154%. Adv. Mater. **34**(18), 2270132 (2022). 10.1002/adma.202270132 10.1002/adma.202109785 35245396

[9] J. Wei, J. Li, C. Duan, L. Yuan, S. Zou et al., High efficiency near-infrared perovskite light emitting diodes with reduced rolling-off by surface post-treatment. Small **19**(20), 2207769 (2023). 10.1002/smll.202207769 10.1002/smll.202207769 36799192

[10] J. Luo, J. Li, L. Grater, R. Guo, A.R. Mohd Yusoff, E. Sargent, J. Tang, Vapour-deposited perovskite light-emitting diodes. Nat. Rev. Mater. **9**(4), 282–294 (2024). 10.1038/s41578-024-00651-8

[11] P. Du, J. Li, L. Wang, L. Sun, X. Wang et al., Efficient and large-area all vacuum-deposited perovskite light-emitting diodes via spatial confinement. Nat. Commun. **12**(1), 4751 (2021). 10.1038/s41467-021-25093-6 34362915 10.1038/s41467-021-25093-6 PMC8346511

[12] L. Wang, J. Xu, J. Luo, W.W. Yu, Thermally evaporated perovskite light-emitting diodes for wide-color-gamut displays in AR/VR devices. Device **2**(10), 100549 (2024). 10.1016/j.device.2024.100549

[13] Z. Zhan, Z. Liu, J. Du, S. Huang, Q. Li et al., Thermally evaporated MAPbBr_3_ perovskite random laser with improved speckle-free laser imaging. ACS Photonics **10**(9), 3077–3086 (2023). 10.1021/acsphotonics.3c00435

[14] Y. Hu, Q. Wang, Y.-L. Shi, M. Li, L. Zhang et al., Vacuum-evaporated all-inorganic cesium lead bromine perovskites for high-performance light-emitting diodes. J. Mater. Chem. C **5**(32), 8144–8149 (2017). 10.1039/C7TC02477K

[15] X. Lian, X. Wang, Y. Ling, E. Lochner, L. Tan et al., Light emitting diodes based on inorganic composite halide perovskites. Adv. Funct. Mater. **29**(5), 1807345 (2019). 10.1002/adfm.201807345

[16] C. Chen, T.-H. Han, S. Tan, J. Xue, Y. Zhao et al., Efficient flexible inorganic perovskite light-emitting diodes fabricated with CsPbBr_3_ emitters prepared via low-temperature in situ dynamic thermal crystallization. Nano Lett. **20**(6), 4673–4680 (2020). 10.1021/acs.nanolett.0c01550 32437162 10.1021/acs.nanolett.0c01550

[17] C.-A. Hsieh, G.-H. Tan, Y.-T. Chuang, H.-C. Lin, P.-T. Lai et al., Vacuum-deposited inorganic perovskite light-emitting diodes with external quantum efficiency exceeding 10% via composition and crystallinity manipulation of emission layer under high vacuum. Adv. Sci. **10**(10), 2206076 (2023). 10.1002/advs.202206076 10.1002/advs.202206076 PMC10074115 36748267

[18] J. Li, P. Du, S. Li, J. Liu, M. Zhu et al., High-throughput combinatorial optimizations of perovskite light-emitting diodes based on all-vacuum deposition. Adv. Funct. Mater. **29**(51), 1903607 (2019). 10.1002/adfm.201903607

[19] B. Dänekamp, N. Droseros, F. Palazon, M. Sessolo, N. Banerji et al., Efficient photo- and electroluminescence by trap states passivation in vacuum-deposited hybrid perovskite thin films. ACS Appl. Mater. Interfaces **10**(42), 36187–36193 (2018). 10.1021/acsami.8b13100 30251819 10.1021/acsami.8b13100

[20] J. Borchert, R.L. Milot, J.B. Patel, C.L. Davies, A.D. Wright et al., Large-area, highly uniform evaporated formamidinium lead triiodide thin films for solar cells. ACS Energy Lett. **2**(12), 2799–2804 (2017). 10.1021/acsenergylett.7b00967

[21] D. Lin, Y. Gao, T. Zhang, Z. Zhan, N. Pang et al., Vapor deposited pure α-FAPbI_3_ perovskite solar cell *via* moisture-induced phase transition strategy. Adv. Funct. Mater. **32**(48), 2208392 (2022). 10.1002/adfm.202208392

[22] M. Kroll, S.D. Öz, Z. Zhang, R. Ji, T. Schramm et al., Insights into the evaporation behaviour of FAI: material degradation and consequences for perovskite solar cells. Sustain. Energy Fuels **6**(13), 3230–3239 (2022). 10.1039/D2SE00373B

[23] A.-F. Castro-Méndez, F. Jahanbakhshi, D.K. LaFollette, B.J. Lawrie, R. Li et al., Tailoring interface energies via phosphonic acids to grow and stabilize cubic FAPbI_3_ deposited by thermal evaporation. J. Am. Chem. Soc. **146**(27), 18459–18469 (2024). 10.1021/jacs.4c03911 38934577 10.1021/jacs.4c03911 PMC11240563

[24] Z. Yuan, Y. Miao, Z. Hu, W. Xu, C. Kuang et al., Unveiling the synergistic effect of precursor stoichiometry and interfacial reactions for perovskite light-emitting diodes. Nat. Commun. **10**(1), 2818 (2019). 10.1038/s41467-019-10612-3 31249295 10.1038/s41467-019-10612-3 PMC6597563

[25] B.-W. Park, H.W. Kwon, Y. Lee, D.Y. Lee, M.G. Kim et al., Stabilization of formamidinium lead triiodide α-phase with isopropylammonium chloride for perovskite solar cells. Nat. Energy **6**(4), 419–428 (2021). 10.1038/s41560-021-00802-z

[26] J.-W. Lee, D.-H. Kim, H.-S. Kim, S.-W. Seo, S.M. Cho et al., Formamidinium and cesium hybridization for photo- and moisture-stable perovskite solar cell. Adv. Energy Mater. **5**(20), 1501310 (2015). 10.1002/aenm.201501310

[27] M. Saliba, T. Matsui, J.-Y. Seo, K. Domanski, J.-P. Correa-Baena et al., Cesium-containing triple cation perovskite solar cells: improved stability, reproducibility and high efficiency. Energy Environ. Sci. **9**(6), 1989–1997 (2016). 10.1039/C5EE03874J 27478500 10.1039/c5ee03874j PMC4936376

[28] Z. Li, M. Yang, J.-S. Park, S.-H. Wei, J.J. Berry et al., Stabilizing perovskite structures by tuning tolerance factor: formation of formamidinium and cesium lead iodide solid-state alloys. Chem. Mater. **28**(1), 284–292 (2016). 10.1021/acs.chemmater.5b04107

[29] K.A. Elmestekawy, A.D. Wright, K.B. Lohmann, J. Borchert, M.B. Johnston et al., Controlling intrinsic quantum confinement in formamidinium lead triiodide perovskite through Cs substitution. ACS Nano **16**(6), 9640–9650 (2022). 10.1021/acsnano.2c02970 35609245 10.1021/acsnano.2c02970 PMC9245356

[30] L. Gil-Escrig, C. Momblona, M.-G. La-Placa, P.P. Boix, M. Sessolo et al., Vacuum deposited triple-cation mixed-halide perovskite solar cells. Adv. Energy Mater. **8**(14), 1703506 (2018). 10.1002/aenm.201703506

[31] H.B. Lee, R. Sahani, V. Devaraj, N. Kumar, B. Tyagi et al., Complex additive-assisted crystal growth and phase stabilization of α-FAPbI_3_ film for highly efficient, air-stable perovskite photovoltaics. Adv. Mater. Interfaces **10**(2), 2201658 (2023). 10.1002/admi.202201658

[32] B. Guo, R. Lai, S. Jiang, L. Zhou, Z. Ren et al., Ultrastable near-infrared perovskite light-emitting diodes. Nat. Photonics **16**(9), 637–643 (2022). 10.1038/s41566-022-01046-3

[33] G. Rainò, N. Yazdani, S.C. Boehme, M. Kober-Czerny, C. Zhu et al., Ultra-narrow room-temperature emission from single CsPbBr_3_ perovskite quantum dots. Nat. Commun. **13**(1), 2587 (2022). 10.1038/s41467-022-30016-0 35546149 10.1038/s41467-022-30016-0 PMC9095639

[34] D. Bi, C. Yi, J. Luo, J.-D. Décoppet, F. Zhang et al., Polymer-templated nucleation and crystal growth of perovskite films for solar cells with efficiency greater than 21%. Nat. Energy **1**, 16142 (2016). 10.1038/nenergy.2016.142

[35] S. Ding, M. Hao, C. Fu, T. Lin, A. Baktash et al., In situ bonding regulation of surface ligands for efficient and stable FAPbI_3_ quantum dot solar cells. Adv. Sci. **9**(35), 2204476 (2022). 10.1002/advs.202204476 10.1002/advs.202204476 PMC9762318 36316248

[36] R. Lindblad, N.K. Jena, B. Philippe, J. Oscarsson, D. Bi et al., Electronic structure of CH_3_NH_3_PbX_3_ perovskites: dependence on the halide moiety. J. Phys. Chem. C **119**(4), 1818–1825 (2015). 10.1021/jp509460h

[37] Z. Zhu, J. Ma, Z. Wang, C. Mu, Z. Fan et al., Efficiency enhancement of perovskite solar cells through fast electron extraction: the role of graphene quantum dots. J. Am. Chem. Soc. **136**(10), 3760–3763 (2014). 10.1021/ja4132246 24558950 10.1021/ja4132246

[38] H. Wang, X. Zhang, Q. Wu, F. Cao, D. Yang et al., Trifluoroacetate induced small-grained CsPbBr_3_ perovskite films result in efficient and stable light-emitting devices. Nat. Commun. **10**(1), 665 (2019). 10.1038/s41467-019-08425-5 30737389 10.1038/s41467-019-08425-5 PMC6368619

[39] M. Liu, O. Voznyy, R. Sabatini, F.P. García de Arquer, R. Munir et al., Hybrid organic-inorganic inks flatten the energy landscape in colloidal quantum dot solids. Nat. Mater. **16**(2), 258–263 (2017). 10.1038/nmat4800 27842072 10.1038/nmat4800

[40] D. Han, J. Wang, L. Agosta, Z. Zang, B. Zhao et al., Tautomeric mixture coordination enables efficient lead-free perovskite LEDs. Nature **622**(7983), 493–498 (2023). 10.1038/s41586-023-06514-6 37557914 10.1038/s41586-023-06514-6

[41] J. Fu, Q. Xu, G. Han, B. Wu, C.H.A. Huan et al., Hot carrier cooling mechanisms in halide perovskites. Nat. Commun. **8**(1), 1300 (2017). 10.1038/s41467-017-01360-3 29101381 10.1038/s41467-017-01360-3 PMC5670184

[42] S.G. Motti, D. Meggiolaro, S. Martani, R. Sorrentino, A.J. Barker et al., Defect activity in lead halide perovskites. Adv. Mater. **31**(47), 1901183 (2019). 10.1002/adma.201901183 10.1002/adma.201901183 31423684

[43] A.-F. Castro-Méndez, J. Hidalgo, J.-P. Correa-Baena, The role of grain boundaries in perovskite solar cells. Adv. Energy Mater. **9**(38), 1901489 (2019). 10.1002/aenm.201901489

[44] L. Xu, J. Li, B. Cai, J. Song, F. Zhang et al., A bilateral interfacial passivation strategy promoting efficiency and stability of perovskite quantum dot light-emitting diodes. Nat. Commun. **11**(1), 3902 (2020). 10.1038/s41467-020-17633-3 32764550 10.1038/s41467-020-17633-3 PMC7413529

[45] S. He, N. Kumar, H.B. Lee, K.-J. Ko, Y.-J. Jung et al., Tailoring the refractive index and surface defects of CsPbBr_3_ quantum dots* vi*a alkyl cation-engineering for efficient perovskite light-emitting diodes. Chem. Eng. J. **425**, 130678 (2021). 10.1016/j.cej.2021.130678

[46] Y. Miao, Y. Ke, N. Wang, W. Zou, M. Xu et al., Stable and bright formamidinium-based perovskite light-emitting diodes with high energy conversion efficiency. Nat. Commun. **10**(1), 3624 (2019). 10.1038/s41467-019-11567-1 31399580 10.1038/s41467-019-11567-1 PMC6689020

[47] Y. Ji, Q. Zhong, M. Yu, H. Yan, L. Li et al., Amphoteric chelating ultrasmall colloids for FAPbI_3_ nanodomains enable efficient near-infrared light-emitting diodes. ACS Nano **18**(11), 8157–8167 (2024). 10.1021/acsnano.3c11941 38456777 10.1021/acsnano.3c11941

