# Supplementary material for: Efficient Thermally Evaporated Near-Infrared Perovskite Light-Emitting Diodes via Phase Regulation
Source: Nanomicro Lett. 2025 May 22;17:270. doi: 10.1007/s40820-025-01776-3 (PMC12098251; doi:10.1007/s40820-025-01776-3)
Supplement: Supplementary file 1 — Supplementary file1 (DOCX 1553 KB) [file 40820_2025_1776_MOESM1_ESM.docx]

Supporting Information for

**Efficient Thermally-Evaporated Near-Infrared Perovskite Light-Emitting Diodes via Phase Regulation**

Siwei He^1,#^, Lanxin Qin^2,#^, Zhengzheng Liu^3,*^, Jae-Wook Kang^4^, Jiajun Luo^2,*^, Juan Du^1,*^

^1^School of Physics and Optoelectronic Engineering, Hangzhou Institute for Advanced Study, University of Chinese Academy of Sciences, Hangzhou 310024, P. R. China.

^2^Wuhan National Laboratory for Optoelectronics (WNLO) and School of Optical and Electronic Information, Huazhong University of Science and Technology (HUST), 1037 Luoyu Road, Wuhan, Hubei 430074, P. R. China

^3^State Key Laboratory of High Field Laser Physics and CAS Center for Excellence in Ultra-Intense Laser Science, Shanghai Institute of Optics and Fine Mechanics, Chinese Academy of Sciences, Shanghai 201800, P. R. China

^4^Department of Flexible and Printable Electronics, LANL-JBNU Engineering Institute-Korea, Jeonbuk National University, Jeonju 54896, Republic of Korea.

^#^Siwei He and Lanxin Qin contributed equally to this work.

*Corresponding authors. E-mail: [du@ucas.ac.cn](mailto:du@ucas.ac.cn) (Juan Du); [luojiajun@hust.edu.cn](mailto:luojiajun@hust.edu.cn) (Jiajun Luo); [liuzhengzheng@siom.ac.cn](mailto:liuzhengzheng@siom.ac.cn) (Zhengzheng Liu)

**S1 Digital images of perovskite thin films**


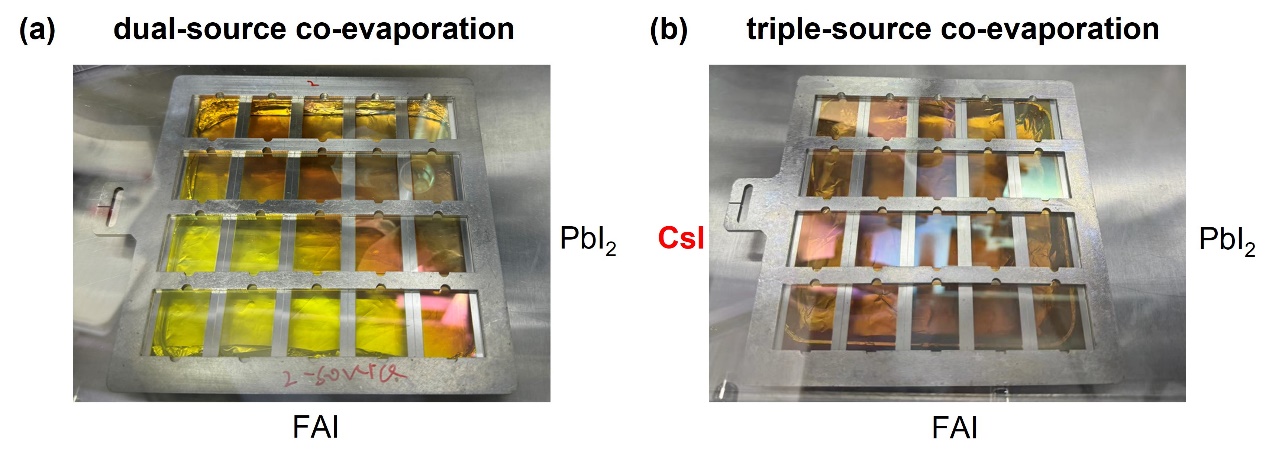


**Fig. S1** Photographs of as-deposited (**a**) FAPbI_3_ via dual-source co-evaporation, and (**b**) FACsPbI_3_ via triple-source co-evaporation

**S2 TRPL analysis**

The PL lifetime decay curves of perovskite films were fitted using bi-exponential function through **Equation S1**:

$I=\sum_{n} A_{n}\exp\left( -\frac{t}{\tau_{n}} \right) n=1, 2, 3\ldots$ (**S1)**

The average PL lifetime (*τ*_avg_) can be determined via **Equation S2**:

$\tau_{\mathrm{avg}}=\frac{\sum_{n} A_{n}\tau_{n}^{2}}{\sum_{n} A_{n}\tau_{n}} n=1, 2, 3\ldots$ (**S2)**

**Table S1** PL decay lifetime of FAPbI_3_ and FACsPbI_3_ based on bi-exponential decay fitting of TRPL spectra

| **Samples** | **A_1_** | ***τ*_1_**  **(ns)** | **A_2_** | **τ_2_**  **(ns)** | **τ_avg_**  **(ns)** |
| --- | --- | --- | --- | --- | --- |
| **FAPbI_3_** | 0.41 | 8.50 | 0.10 | 68.83 | 48.55 |
| **FACsPbI_3_** | 1.90 | 4.84 | 0.18 | 80.37 | 51.02 |

**S3 XPS analysis**


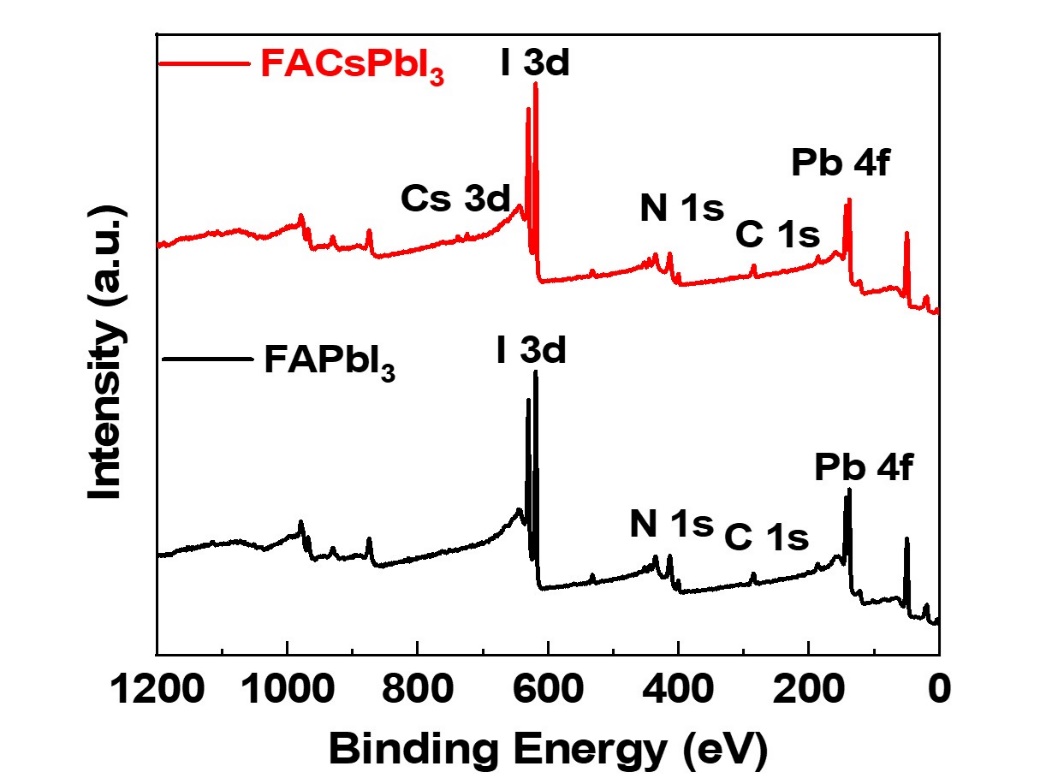


**Fig. S2** Overall XPS spectra of FAPbI_3_ and FACsPbI_3_ thin film

**S4 UPS analysis**


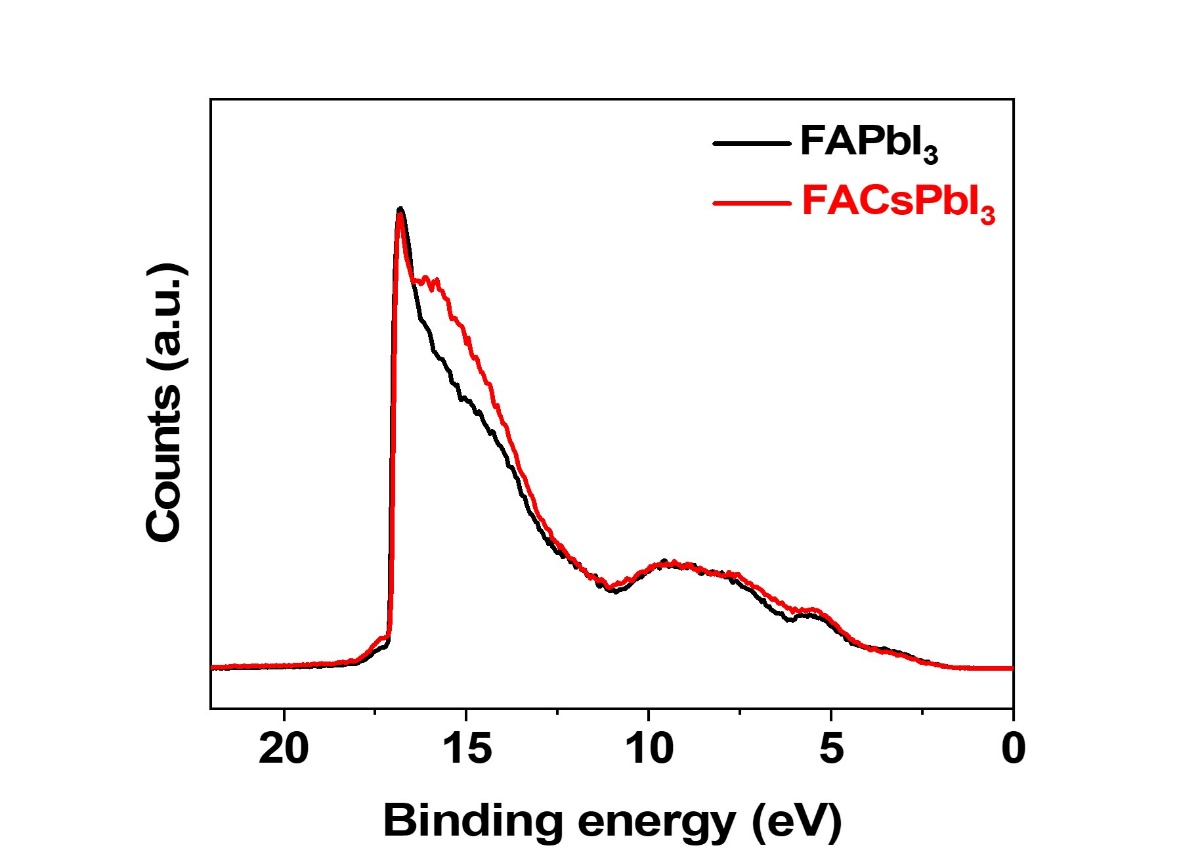


**Fig. S3** UPS spectra of FAPbI_3_ and FACsPbI_3_ thin films

The valence band maximum (*E*_VBM_) of the perovskite films were calculated using **Equation S3**:

*E*_VBM_ = *hν* - (*E*_cut-off_ - *E*_onset_) (**S3)**

where *hν* is the ultraviolet radiation energy (21.22 eV).

The Fermi levels of the perovskite films were calculated using **Equation S4:**

*E*_Fermi_ =*hv* - *E*_cut-off_ **(S4)**

The conduction band minimum (*E*_CBM_) of the perovskite films were calculated using **Equation S5**:

*E*_CBM_ = *E*_VBM_ - *E*_g_ (**S5)**

**Table S2** Detailed band energy derived from UPS spectra for FAPbI_3_ and FACsPbI_3_ thin films

| **Samples** | ***E*_cut-off_ [eV]** | ***E*_on-set_**  **[eV]** | ***E*_VBM_ [eV]** | ***E*_Fermi_ [eV]** | ***E*_g_**  **[eV]** | ***E*_CBM_ [eV]** |
| --- | --- | --- | --- | --- | --- | --- |
| **FAPbI_3_** | 17.11 | 1.58 | -5.69 | -4.11 | 1.60 | -4.09 |
| **FACsPbI_3_** | 17.06 | 1.50 | -5.66 | -4.16 | 1.54 | -4.12 |

**S5 Morphologies**


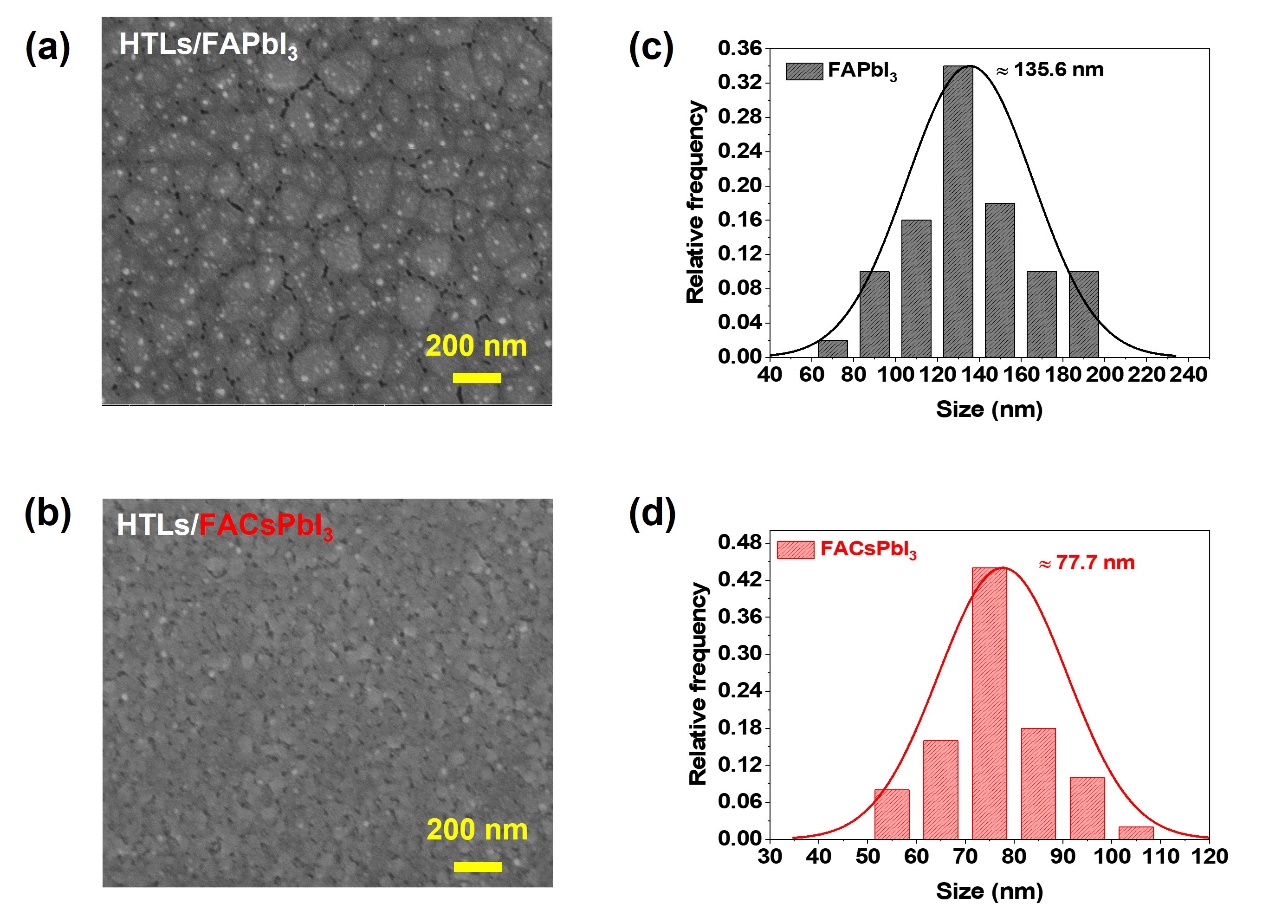


**Fig. S4** FE-SEM images of (**a**) FAPbI_3_ and (**b**) FACsPbI_3_. The crystal size distribution of (**c**) FAPbI_3_, and (**d**) FACsPbI_3_ deposited on PEDOT:PSS/TFB hole transport layer

**S6 FIB images**


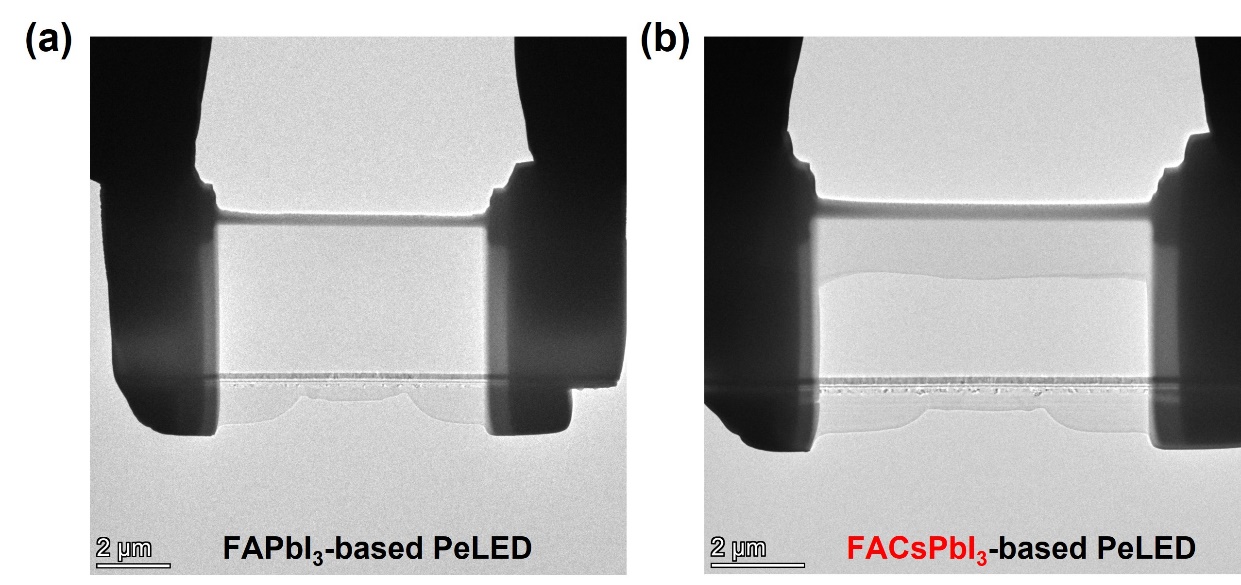


**Fig. S5** FIB images of the FAPbI_3_ and FACsPbI_3_ for the characterization of STEM

**S7 Cross-sectional and EDS images**


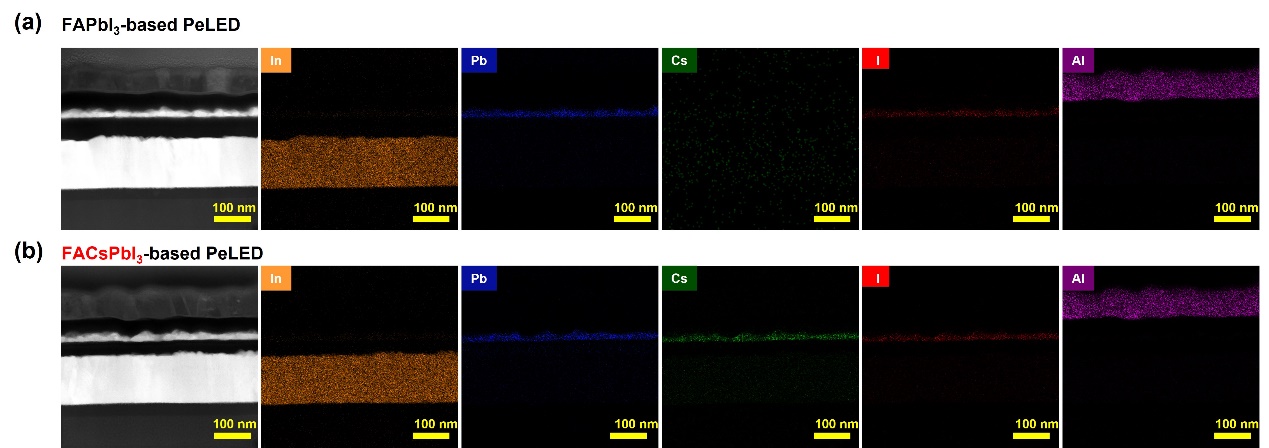


**Fig. S6** Cross-sectional image and EDS spectra of (**a**) FAPbI_3_-based, and (**b**) FACsPbI_3_-based PeLEDs

**S8 EQE and wavelength peak distribution**


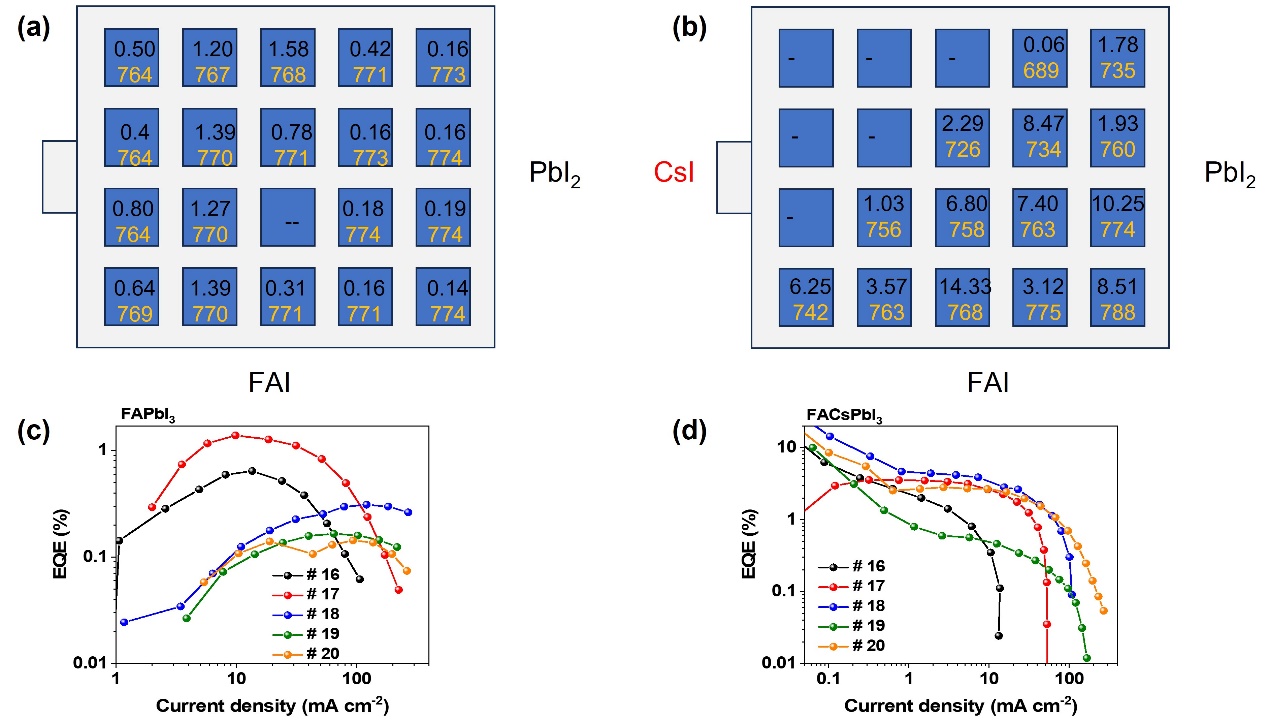


**Fig. S7** EQE values (black, upper data) and wavelength peaks (yellow, lower data) for (**a**) FAPbI_3_ via dual-source co-evaporation, and (**b**) FACsPbI_3_ via triple-source co-evaporation. EQE-current density from the fourth row (denoted as #16-20) for (**c**) FAPbI_3_-based and (**d**) FACsPbI_3_-based devices

**S9 SCLC analysis**


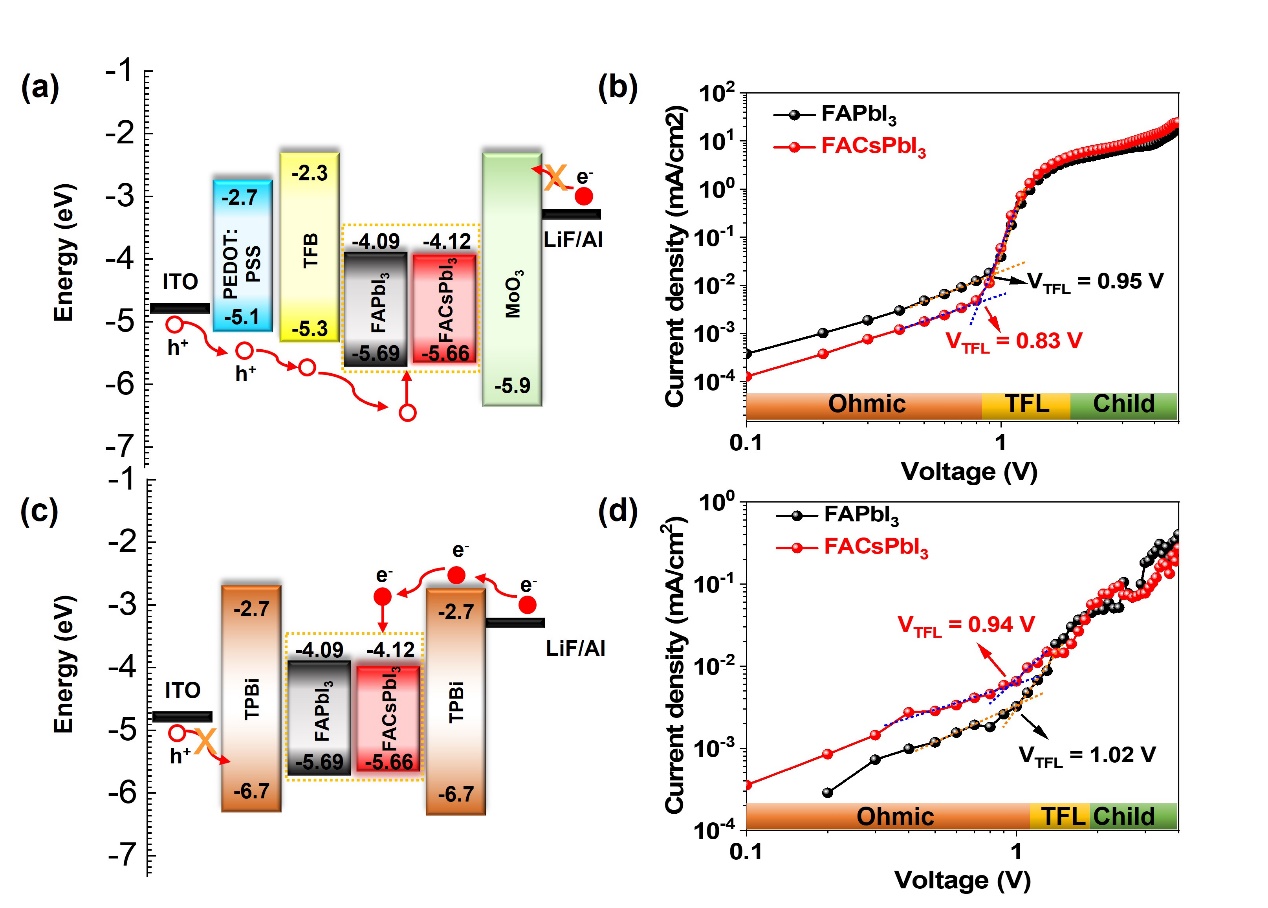


**Fig. S8** (**a**) Energy band diagram, and (**b**) current density-voltage curves for the hole-only devices based on FAPbI_3_ and FACsPbI_3_. (**c**) Energy band diagram, and (**d**) current density-voltage curves based on FAPbI_3_ and FACsPbI_3_

**Table S3** Performance of the state-of-the-art NIR-PeLEDs

| **Deposition method of perovskite** | **Perovskite** | **Device structure** | **EL**  **(nm)** | **EQE**  **(%)** | **Radiance**  **(W sr^-1^ m^-2^)** | **Year** | **Refs.** |
| --- | --- | --- | --- | --- | --- | --- | --- |
| **Thermal evaporation** | FACsPbI_3_ | ITO/PEDOT:PSS/ TFB/Pe/TPBi/LiF/Al | 770 | 10.25 | 2.64 | 2024 | This work |
|  | MAPbI_3_ | ITO/PEDOT:PSS/poly-TPD/Pe/PCBM/Ba/Ag | 765 | 0.04 | 0.084 | 2014 | [S1] |
|  | MAPbI_3_ | ITO/PEDOT:PSS/poly-TPD/Pe/PCBM/Ba/Ag | 765 | 0.06 | N.A. | 2015 | [S2] |
|  | MAPbI_3_ | ITO/MeO-TPD:F4TCNQ/MeO-TPD/Pe/Bphen/Bphen:Cs/Ag | 768 | 0.35 | 4.2 | 2016 | [S3] |
|  | MAPbI_3_ | ITO/ C_60_/Pe/ TaTm /Au | 760 | 1.92 | 4.1 | 2018 | [S4] |
| **Solution-based process** | MAPbI_3-x_Cl_x_ | ITO/TiO_2_/Pe/F8/MoO_3_/Ag | 754 | 0.76 | 13.2 | 2014 | [S5] |
|  | FPMAI-MAPb_0.6_Sn_0.4_I_3_ | ITO/Poly-TPD/Pe/TPBi/LiF/Al | 917 | 5.0 | 2.7 | 2018 | [S6] |
|  | Cs_0.17_FA_0.83_PbI_2.5_Br_0.5_ | ITO/ZnO/PEIE/Pe/TFB/MoO_3_/Au | 800 | 17.4 | 965 | 2020 | [S7] |
|  | KI-MAPb_0.8_Sn_0.2_I_3_ | ITO/Poly-TPD/Pe/TPBi/LiF/Al | 868 | 9.6 | 0.025 | 2022 | [S8] |
|  | PEAI-FAPbI_3_ QDs | ITO/PEDOT:PSS/VB-FNPD/Pe/CN-T2T/LiF/Al | 772 | 15.4 | 128.1 | 2022 | [S9] |
|  | SFB10-FAPbI_3_ | ITO/ZnO/PEIE/Pe/TFB/MoOx/Au | 803 | 22.8 | 278.9 | 2022 | [S10] |
|  | Yb^3+^ :CsPb(Cl_1-x_Br_x_)_3_ QDs | ITO/PEDOT:PSS/Poly-TPD/PVK/Pe/TPBi/Liq/Al | 990 | 7.7 | NA | 2023 | [S11] |
|  | MSPE-FAPbI_3_ | ITO/ZnO/PEIE/Pe/Poly-TPD/MoO_x_/Au | 800 | 23.8 | > 3200 | 2023 | [S12] |
|  | Yb^3+^:CsPb(Cl_1−x_Br_x_)_3_ QDs | ITO/PEDOT:PSS/poly-TPD/PVK/Pe/PO-T2T/Liq/Al | 990 | 8.5 | 52.5 | 2024 | [S13] |
|  | CsSnI_3_ | ITO/PEDOT:PSS/Pe/TPBi/LiF/Al | 948 | 2.63 | 226 | 2024 | [S14] |

**Supplementary References**

[S1] L. Gil-Escrig, G. Longo, A. Pertegás, C. Roldán-Carmona, A. Soriano et al, Efficient photovoltaic and electroluminescent perovskite devices. Chem. Comm. **51**, 569–571 (2015). <https://doi.org/10.1039/c4cc07518h>

[S2] L. Gil-Escrig, A. Miquel-Saempere, M. Sessolo, H.J. Bolink, Mixed iodide-bromide methylammonium lead perovskite-based diodes for light emission and photovoltaics. J. Phys. Chem. Lett. **6**, 3743–3748 (2015). <https://doi.org/10.1021/acs.jpclett.5b01716>

[S3] A. Genco, F. Mariano, S. Carallo, V.L.P. Guerra, S. Gambino et al, Fully vapor-deposited heterostructured light-emitting diode based on organo-metal halide perovskite. Adv. Electron Mater. **2,** 1500325 (2016). <https://doi.org/10.1002/aelm.201500325>

[S4] B. Dänekamp, N. Droseros, F. Palazon, M. Sessolo, N. Banerji et al, Efficient photo- and electroluminescence by trap states passivation in vacuum-deposited hybrid perovskite thin films. ACS Appl. Mater. Interfaces **10**, 36187–36193 (2018). <https://doi.org/10.1021/acsami.8b13100>

[S5] Z.K. Tan, R.S. Moghaddam, M.L. Lai, P. Docampo, R. Higler et al, Bright light-emitting diodes based on organometal halide perovskite. Nat. Nanotechnol. **9** 687–692 (2014). <https://doi.org/10.1038/nnano.2014.149>

[S6] W. Qiu, Z. Xiao, K. Roh, N.K. Noel, A. Shapiro et al, Mixed lead–tin halide perovskites for efficient and wavelength-tunable near-infrared light-emitting diodes. Adv. Mater. **31,** 1806105 (2019). <https://doi.org/10.1002/adma.201806105>

[S7] Y.H. Jia, S. Neutzner, Y. Zhou, M. Yang, J.M.F. Tapia et al, Role of excess FAI in formation of high-Efficiency FAPbI_3_-based light-emitting diodes, Adv. Funct. Mater. **30,** 1906875 (2020). <https://doi.org/10.1002/adfm.201906875>

[S8] H. Yu, W. Chen, Z. Fang, L. Ding, B. Cao et al, Alkalis-doping of mixed tin-lead perovskites for efficient near-infrared light-emitting diodes, Sci. Bull. (Beijing) **67**, 54–60 (2022). <https://doi.org/10.1016/j.scib.2021.07.021>

[S9] Z.L. Tseng, L.C. Chen, L.W. Chao, M.J. Tsai, D. Luo et al, Aggregation control, surface passivation, and optimization of device structure toward near-infrared perovskite quantum-dot light-emitting diodes with an EQE up to 15.4%, Adv. Mater. **34,** 2109785 (2022). <https://doi.org/10.1002/adma.202109785>

[S10] B. Guo, R. Lai, S. Jiang, L. Zhou, Z. Ren et al, Ultrastable near-infrared perovskite light-emitting diodes, Nat. Photonics **16**, 637–643 (2022). <https://doi.org/10.1038/s41566-022-01046-3>

[S11] Y.J. Yu, C. Zou, W.S. Shen, X. Zheng, Q.S. Tian et al, Efficient near-infrared electroluminescence from lanthanide-doped perovskite quantum cutters, Angew. Chem. Int. Ed. **62,** 2302005 (2023). <https://doi.org/10.1002/anie.202302005>

[S12] Y. Sun, L. Ge, L. Dai, C. Cho, J. Ferrer Orri et al, Bright and stable perovskite light-emitting diodes in the near-infrared range, Nature **615**, 830–835 (2023). <https://doi.org/10.1038/s41586-023-05792-4>

[S13] J.L. Pan, W.S. Shen, S.N. Li, Z. Da Zhang, F. Zhao et al, Polarity-mediated antisolvent control enables efficient lanthanide-based near-Infrared perovskite LEDs, Nano Lett. **24**, 2765–2772 (2024). <https://doi.org/10.1021/acs.nanolett.3c04586>

[S14] F. Yuan, G. Folpini, T. Liu, U. Singh, A. Treglia et al, Bright and stable near-infrared lead-free perovskite light-emitting diodes, Nat. Photonics **18**, 170–176 (2024). <https://doi.org/10.1038/s41566-023-01351-5>
